# Using the Kolb’s experiential learning cycle to explore the extent of application of one health competencies to solving global health challenges; a tracer study among AFROHUN-Uganda alumni

**DOI:** 10.1186/s12992-022-00841-5

**Published:** 2022-05-12

**Authors:** Tonny Ssekamatte, John Bosco Isunju, Aisha Nalugya, Richard K. Mugambe, Patrick Kalibala, Angella Musewa, Winnie Bikaako, Milly Nattimba, Arnold Tigaiza, Doreen Nakalembe, Jimmy Osuret, Solomon Tsebeni Wafula, Esther Buregyeya, Fatima Tsiouris, Susan Michaels-Strasser, John David Kabasa, William Bazeyo

**Affiliations:** 1 Department of Disease Control and Environmental Health, School of Public Health, Kampala, College of Health Sciences, Makerere University, P.O Box 7072, Kampala, Uganda; 2 Africa One Health University Network, Plot 20B Kawalya Kagwa Cl, Kololo, Kampala, Uganda; 3 Makerere University College of Veterinary Medicine, Animal Resources and Biosecurity, Kampala, Uganda; 4 Columbia University, Columbia University Mailman School of Public Health, New York, NY USA

**Keywords:** One health, Tracer study, Competences, Global health challenges, Experiential learning

## Abstract

**Background:**

The Africa One Health University Network (AFROHUN) with support from the United States Agency for International Development (USAID), has since 2012 conducted pre and in-service One health (OH) trainings with the objective of improving global health security. These trainings aim to build competencies that, enhance a multidisciplinary approach to solving global health challenges. Despite the investment in OH trainings, there is limited documentation of the extent of acquisition and application of the OH competencies at workplaces. This tracer study explored the extent of acquisition and application of the OH competencies by the AFROHUN-Uganda alumni.

**Methods:**

A cross-sectional study was conducted among a random sample of 182 AFROHUN-Uganda alumni of 2013–2018 cohorts. A blended approach of interviewer-administered and self-administered questionnaires was used. Virtual platforms such as Zoom, Microsoft teams, and Skype, and phone interviews were used to collect data when face-to-face interactions with alumni were not possible. Data were collected electronically, either through a link or with the aid of the KoboCollect mobile application, pre-installed on android enabled devices, and analysed using STATA14.0.

**Results:**

The majority of respondents, 78.6% (143/182) had jobs that required application of OH knowledge and skills, 95.6% (174/182) had learned employable skills from OH activities and 89.6% (163/182) had applied such skills when searching for employment. About 21.7% (34/180) to a very high extent required OH field-specific theoretical knowledge at their workplaces, 27.4% (43/80) to a very high extent required OH field-specific practical knowledge/skills, 42.7% (67/180) to a high extent required a change in attitude and perceptions towards working with people from different disciplines, 49.0% (77/180) required collaboration and networking skills, and more than half, 51.0% (80/180) required team building skills.

**Conclusions:**

The majority of OH alumni to a very high extent acquired and applied OH competences such as teamwork, effective communication, community entry and engagement, report writing and problem-solving skills. This study revealed the significant contribution of the AFROHUN Uganda OH activities towards supportive work environments, and highlights areas of improvement such as supporting the trainees to acquire people-management skills, innovation, and an entrepreneurial mind set.

## Background

The increase in global health challenges is linked to the close interaction between humans, animals and the environment [1]. This close interaction is often precipitated by population pressure, urbanization, advances in international travel, trade and animal industry, deforestation, climate change, and encroachment on wildlife habitats [2]. The human-animal and environment interaction has led to the international spread of emerging and re-emerging infectious diseases, including Coronavirus disease (COVID-19), and hemorrhagic fevers such as Ebola virus disease (EVD) and Marburg [3, 4]. Emerging and re-emerging infectious disease outbreaks and spillover events have become the new norms [2]. These events are exacerbated by frequent exposure of humans to disease vectors and reservoirs, antimicrobial resistance, weak surveillance systems, and limited laboratory diagnostic capacity [5–7].

The One Health (OH) approach is increasingly being recognised as a practical and innovative way of addressing the myriad of twenty-first-century global health challenges [2, 7]. The OH approach is a collaborative, multisectoral, and transdisciplinary approach in which stakeholders at local, regional, national, and global levels work together to achieve optimal health for humans, animals and their shared environment [8]. This approach is increasingly being used in prediction, detection, prevention and response to global health challenges such as outbreaks of zoonotic diseases and antimicrobial resistance [9]. It brings together public health professionals, veterinarians, agricultural scientists, anthropologists, economists, educators, engineers, entomologists, epidemiologists, hydrologists, microbiologists, nutritionists, physicians, and sociologists among other cadres to work collaboratively to attain human, animal and environmental health [10].

The OH approach strives to improve global health security by building a multidisciplinary health workforce. Attainment of the goals of the OH approach is guided by the Berlin Principles, an update of the 2004 Manhattan Principles, which first coined the term One Health for the broader public [2]. Berlin Principle number 10 calls upon world leaders, governments, civil society, the global health and conservation communities, academia and scientific institutions, business, finance leaders, and investment holders to educate and create awareness of OH among the global citizenship. It emphasises the need to sensitise children and adults in schools, communities and universities on OH, and to influence policy to ensure the interdependences between human, animal and environmental health [2, 11].

The tenth Berlin Principle also recognises that the acquisition and application of OH competencies is relevant to promote OH, making it imperative to understand how these competencies are being mobilized to solve global health challenges. There is consensus among practitioners that the multidisciplinary and transdisciplinary, and collaborative nature of OH requires specific knowledge, skills and competencies, processes and institutions that facilitate policy and operations to be co-managed and co-delivered across jurisdictions [12]. OH competencies encompass the knowledge, behaviour, and skills needed to achieve desired results within the One Health paradigm [13, 14]. Having the required knowledge, skills and competencies enables OH practitioners to counter global barriers that exist because of disciplinary silos [15–17]. The major skills and competence domains required by OH practitioners have previously been documented [14, 18–20], and include management, communication and informatics, values and ethics, leadership, teamwork and collaboration, and roles and responsibilities.

Although vital, OH competencies required to build successful teams and programs are often overlooked. Most of the discussion on developing the OH workforce focuses on creating cross-disciplinary awareness and technical skills [12] rather than soft skills such as leadership, communication and informatics, systems thinking and management among others [18, 20, 21]. To address this challenge, the Africa One Health University Network (AFROHUN), through its One Health Workforce (OHW) and One Health Workforce next-generation projects undertook competency-based training to enhance the knowledge and skill set of the pre- and in-service workforce in its network of 10 member countries, 27 institutions and 19 universities [1, 3].

AFROHUN uses a more integrative and dynamic education system to match global health needs and to produce a workforce that can effectively and efficiently predict, detect and respond to complex global health challenges [15]. To achieve this, AFROHUN developed 16 training modules that aim to address the training needs of different sectors and disciplines [22]. The modules emphasize teamwork, community engagement, research and effective communication across disciplines, among other competencies [22]. The OH trainings incorporate both theoretical and experiential learning with the view of equipping the workforce with the basic technical and non-technical skills and competencies that can complement their specific areas of expertise necessary for OH practitioners, regardless of discipline [15].

In order to build a more sustainable workforce, AFROHUN together with partner institutions of higher learning provides scholarships, graduate fellowships and residencies. AFROHUN, through the OH clubs, provides students with an opportunity to engage in outbreak investigation. In addition, through the OH Institute, it conducts OH field placements in which students of the various disciplines live and work together in small multidisciplinary teams in the community [15]. Together with the different professionals in the field, supervisors and community members, the multi-disciplinary team of students once placed, embarks on community assessments to identify key health problems in the communities of placement [23]. This is followed by the implementation of activities aiming at solving these challenges as well as routine monitoring and evaluation.

While numerous OH programs/training and research activities have been carried out among academic, non-academic, government, corporate, and non-profit entities [24], there has been little progress in understanding the extent of acquisition and application of the OH competencies. This tracer study was based on Kolb’s experiential Learning Cycle [25] and the systems theory framework (STF) of career development [26] to explore the extent to which the AFROHUN-Uganda alumni acquired and applied the OH competencies. Kolb’s cycle suggests that effective learning is achieved when a participant obtains a concrete experience of the subject matter followed by (2) observation of and reflection on that experience which leads to (3) the formation of abstract concepts (analysis) and generalizations (conclusions) which are then (4) used to test hypothesis in future situations, resulting in new work experiences. The STF of career development suggests that career development is a dynamic process, depicted through its process influences, change over time and chance [27].

## Methods

### Study design, setting and population

A tracer study utilizing quantitative data collection methods was used to explore the extent of acquisition and application of OH competencies among AFROHUN-Uganda alumni from the 2013–2018 cohorts of Makerere University and Mbarara University of Science and Technology. Since its inception, AFROHUN-Uganda tracks its alumni to ascertain their employment status, assess whether the training they received meets the market demands and inform the training programmes. To achieve the goals of alumni tracking and engagement, the organisation has an individual (TS) who keeps track of the whereabouts of the alumni and progression in their careers. Data analysed in the current paper is, therefore, based on an alumni tracking and engagement activity. Tracer studies are surveys of a homogenous group of students/trainees that are conducted some period after graduation or at the end of training to assess their course of study, the transition to work, employment status, career and application of learned competencies [28]. These studies have been conducted previously and can be used in settings where there’s limited evidence of employment outcomes of training graduates [29] as is the case with the AFROHUN-Uganda alumni.

### Description of OH activities

AFROHUN developed a OH Institute (OHI) intended to train and transform the knowledge of young interdisciplinary teams of undergraduate and graduate students, in detection, prevention and response to global health challenges, including infectious disease threats. In the OHI, students are engaged through didactic instruction on the theoretical principles of OH [22]. The OH theoretical principles were explored through 16 training modules designed to address the needs of different sectors and disciplines in managing OH challenges and were delivered by a multidisciplinary team of faculty [1, 22]. The multidisciplinary delivery model involved group discussions, role-plays, case studies and simulations [1]. This was followed by experiential learning through undergraduate field placements and graduate fellowships [1, 22]. The undergraduate field attachments involved attaching students to predetermined demo sites which gave them an opportunity to apply the knowledge acquired through theoretical classes. At the demo site, the students embarked on problem identification, prioritization and implementation of multidisciplinary solutions in teams under the supervision of different professionals in the field and community members [23]. On the other hand, multidisciplinary fellowship placements for graduate students used a mentorship-training model. Graduate students were placed at selected partner organizations and assigned two mentors (academic and field) to support and guide them to acquire OH competencies, such as problem-solving, multisectoral communication, community engagement, proposal writing, scientific writing and publication. The small grants, which were offered to both undergraduate and graduate students supported research and innovations specific to addressing OH challenges. Conversely, the OH student club is a multidisciplinary team of students in Makerere University which has helped participants develop skills and competencies in OH leadership, collaboration and teamwork, community engagement, research, innovation and scientific communication through innovative intellectual debate and engagement around identified OH challenges. There was no difference in the implementation of the 2013–2015 and 2016–2018 OH editions.

### History of development of OH competencies

This study adopted the definition of OH core competencies used by Frankson, Hueston [18]. Frankson, Hueston [18] describe OH competencies as the knowledge, skills, and attitudes required to be effective within a OH workforce. Identification of OH competencies dates back to 2008 and 2011, a period in which the Bellagio Working Group, Stone Mountain Meeting Training Workgroup and USAID/RESPOND initiative independently identified the knowledge, skills and behaviours required by OH practitioners [18]. The major OH competence domains identified by these working groups included management, communication and informatics, values and ethics, leadership, teamwork and collaboration, roles and responsibilities, and systems thinking [18]. Following international efforts to identify OH competencies, the RESPOND One Health Core Competency (OHCC) initiative which involved the One Health Central and East Africa (OHCEA) (now known as AFROHUN), the South East Asia One Health University Network (SEAOHUN) along with U.S. partner organizations developed a competency framework to inform the design of OH curricula [30]. The major domains in this competency framework included management, communications and informatics, culture and beliefs, leadership, collaboration and partnerships, values and ethics, and systems thinking [30, 31]. In 2019, AFROHUN used the OH competency framework to develop 8 core competencies and 8 technical modules which were delivered to alumni. The modules related to improving the competencies of OH practitioners included 1) one health systems thinking, 2) gender analysis in one health 3) one health risk communication, 4) leadership, 5) collaboration and partnerships, 6) management, 7) culture, beliefs, values and ethics, and policy and advocacy [31].

### Defining OH competencies

According to the AFROHUN OH competency-based curriculum, systems thinking allows OH practitioners to solve “wicked complex problems” through a simplified process [31]. It provides an avenue to analyse the human-animal-environmental interactions and the different disciplines engaged and how they work together as a system to solve complex health problems. One Health Risk Communication is defined as the use of a mix of communication and engagement strategies and tactics, including but not limited to, media communications, social media, mass awareness campaigns, health promotion, stakeholder engagement, social mobilization and community engagement. Leadership has been defined as a process of social influence, which maximizes the efforts of others, towards the achievement of a goal [32]. Collaboration entails the mutual engagement of different stakeholders in a coordinated effort to solve a problem together [33] while a partnership is an arrangement where parties agree to cooperate to advance their mutual interests. It involves sharing both losses and profits accrued from their engagement [34]. Management is about administration and controlling the operations and resources of an entity or an organisation [34]. Culture refers to the systems of knowledge (including rituals and practices) shared by a relatively large group of people [35, 36] while ethics refers to the moral principles that govern a person’s behaviour. Ethics also refers to standards of conduct or behaviour that distinguish between right/wrong, and good/bad [37]. Policy and advocacy in the context of the AFROHUN-Uganda OH training entailed building the capacity of the alumni to develop, analyse and implement OH related policies [31].

### Sample size and sampling technique

The sample size was estimated using the Kish Leslie formula for cross-sectional studies [38]. We assumed the proportion of AFROHUN-Uganda alumni who applied OH competencies at their workplaces at 50%, a 5% margin of error and the standard normal deviation at 95% (1.96), giving a sample size of 384. Since the total number of alumni is only 308, with the calculated sample size higher by over 5% of the total population, we applied the sample size formula by Daniels (1999) and obtained 170. Considering a non-response rate of 10%, a total sample size of 189 alumni was obtained.

A simple random sampling technique was used in the selection of the respondents. First, a list of the alumni containing their respective email addresses and phone numbers was obtained from the AFROHUN-Uganda country office and their former colleges. We then used the Ms. EXCEL function (RAND formula: =RAND ()) to generate a random sample from that list. In cases where reliable contact details could not be obtained from AFROHUN-Uganda, research assistants requested the respondents to share the contact details of members in their cohort.

### Data collection techniques, tools and study variables

Although face-to-face interviews were considered the core approach to data collection in this study, virtual interviews were also conducted when necessary. Research assistants emailed the link to the e-questionnaires to the prospective respondents that was. Besides, phone and online/internet-based interviews using Skype and Zoom were also conducted, depending on the availability and preference of the respondents. These approaches have been successfully applied in existing tracer studies [39, 40]. We adapted the structured questionnaire used by the National Council for Higher Education (NCHE) to conduct a tracer study of 2005 graduates from five universities and four colleges [40]. We tailored the questions to the different fields, regions and employing organizations/ institutions in the study. The questionnaire obtained data on background characteristics such as the age, sex, nature of AFROHUN-Uganda OH activities attended, year of attendance of the activities, and highest level of academic qualification. In addition, data were collected on the extent to which respondents acquired OH competencies during participation in AFROHUN-Uganda OH activities, OH competencies required in their employing organizations/institutions and application of OH competencies in employing organizations. We used a Likert scale to explore the extent of acquisition and application of the OH competencies. Respondents were asked to what extent they had acquired and applied OH competencies. The responses to these questions were 1) very high extent, 2) high extent, 3) some extent, 4) limited extent and 5) not at all.

### Data management and analysis

Data were collected and entered using Kobo Collect mobile application and synchronized onto the server daily. This allowed for real-time data capture and entry, minimized errors at entry and eased data cleaning. Finally, data were downloaded into Microsoft excel for cleaning. Data cleaning included checking for accuracy, completeness and consistency of data. Data were then exported to Stata version 14.0 software for statistical analysis. Descriptive analyses such as frequencies, proportions, and means (where appropriate) were performed.

### Quality control and assurance

Research assistants (RAs) with a minimum of a Bachelor’s degree in Environmental Health Sciences, Social Sciences and other relevant fields were recruited. The PIs recruited only RAs who were well conversant with English given that it’s was the main language used by our respondents. All RAs were trained on the study protocol, and ethical issues and oriented on the different interviewing techniques to ensure quality data collection. We designed the data entry form with skips and restrictions to ensure quality data entry. The tool was pretested to assess the comprehension and clarity of questions. We also supervised the RAs to ensure that they followed the study protocol and observed ethics during data collection.

## Results

### Background characteristics of the 2013–2018 AFROHUN-Uganda alumni

This study recruited a total of 182 respondents, representing a 96.3% response rate. Less than half, 41.2% (75/182) were females. The mean age of the respondents was 28.7 (SD = 4.6), the median age was 27 while the modular age was 26. The youngest alumnus was 23  while the eldest was 43 years old. Nearly three quarters, 74.7% (136/182) were aged below 30 years. The average age at award of the undergraduate degree was 24.6 (SD = 2.9). Almost three quarters, 73.6% (134/182) had attained a bachelor’s degree, 24.2% (44/182) and 2.2% (4/182) had attained master’s and postgraduate diploma respectively as their highest level of academic qualification (Table 1). About 29.7% (46/155) of the alumni were employed in the health sector, 28.3% (44/155) in research and academia, 15.5% (24/155) in veterinary medicine or wildlife management, 11.6% (18/155) in agriculture, 11.0% (17/155) in trade/ business/ entrepreneurship, 9.7% (15/155) in water, sanitation and hygiene, and 3.9% (6/155) in information communication and technology.

**Table 1:** Background characteristics of the 2013–2018 AFROHUN-Uganda alumni

Variable	Attribute	Frequency *N* = 182 (%)
Sex	Male	107 (58.8)
	Female	75 (41.2)
Age in years	Below 30	136 (74.7)
	30 and above	46 (25.3)
Year of attendance of any AFROHUN-Uganda capacity building program	2013–2015	37 (20.3)
	2016–2018	145 (79.7)
Nature of AFROHUN-Uganda capacity building activities that the alumni participated in^a^	OH field attachment	163 (89.6)
	OH students’ club	8 (4.4)
	Master of Veterinary Public Health and Management	3 (1.6)
	Got a scholarship	2 (1.1)
	Fellowship	22 (12.1)
	Outbreak Investigations	20 (11)
	OH residency	2 (1.1)
	Innovations	11 (6)
Had any other academic qualification prior to the award of the most recent qualification	Yes	22 (12.1)
	No	160 (87.9)
Highest academic qualification	Bachelors	134 (73.6)
	Masters	44 (24.2)
	Post Graduate Diploma	4 (2.2)
Was employed after participation in the AFROHUN-Uganda capacity building programme	Yes	159 (87.4)
	No	23 (12.6)

^a^Multiple response

### Extent to which respondents acquired OH competencies during participation in AFROHUN-Uganda OH activities

Of the 182 participants, 98.9% (180/182) responded on the extent of acquisition of OH competencies. Almost half, 47.8% (86/180) to a high extent acquired field-specific theoretical knowledge and field-specific practical knowledge. About 88.3% (159/180), to a high extent acquired a change in attitude and perception toward working in multidisciplinary teams. Collaboration skills and communication skills were acquired to a high extent by less than half, 43.3% (78/180) and 42.8% (77/180), respectively. More than a third, 42.2% (76/180) and 40.0% (72/180) acquired leadership skills and social skills to a high extent, respectively. About half, 51.1% (92/180) to a high extent acquired team-building skills. Almost half, 43.9% (79/180) acquired planning, coordinating and organizing skills to a high extent (Table 2).

**Table 2:** Extent of acquisition of OH competencies among the 2013–2018 AFROHUN-Uganda alumni

	Extent of acquisition of OH competencies *N* = 180 (n)%				
OH Competencies	Very high	High	Some	Limited	Not at all
Field-specific theoretical knowledge	50 (27.8)	6 (47.8)	40 (22.2)	4 (2.2)	0 (0.0)
Field-specific practical knowledge/skills	59 (32.8)	86 (47.8)	30 (16.7)	5 (2.7)	0 (0.0)
Attitudinal change towards working with multi-disciplinary teams	82 (45.6)	77 (42.8)	17 (9.4)	4 (2.2)	0 (0.0)
Critical thinking skills	50 (27.8)	84 (46.7)	42 (23.3)	4 (2.2)	0 (0.0)
Creativity	46 (25.6)	95 (52.8)	34 (18.9)	4 (2.2)	1 (0.6)
Collaboration across disciplines	62 (34.4)	78 (43.3)	34 (18.9)	5 (2.8)	1 (0.6)
Communication across disciplines	69 (38.3)	77 (42.8)	30 (16.7)	4 (2.2)	0 (0.0)
Leadership skills	59 (32.8)	76 (42.2)	37 (20.6)	8 (4.4)	0 (0.0)
Social (influencing) skills	57 (31.7)	72 (40)	40 (22.2)	11 (6.1)	0 (0.0)
ICT skills	22 (12.2)	39 (21.7)	50 (27.8)	51 (28.3)	18 (10)
Analytical skills	30 (16.7)	78 (43.3)	52 (28.9)	18 (10)	2 (1.1)
Problem solving skills	47 (26.1)	102 (56.7)	23 (12.8)	8 (4.4)	0 (0.0)
Ability to take initiative/lead	50 (27.8)	78 (43.3)	39 (21.7)	13 (7.2)	0 (0.0)
Entrepreneurial skills	25 (13.9)	50 (27.8)	57 (31.7)	36 (20)	12 (6.7)
Team building skills	65 (36.1)	92 (51.1)	17 (9.4)	5 (2.8)	1 (0.6)
People management skills	52 (28.9)	71 (39.4)	46 (25.6)	11 (6.1)	0 (0.0)
Customer orientation skills	52 (28.9)	69 (38.3)	42 (23.3)	11 (6.1)	6 (3.3)
Assertiveness	36 (20.0)	69 (38.3)	51 (28.3)	18 (10)	6 (3.3)
Decisiveness	36 (20.0)	72 (40)	56 (31.1)	11 (6.1)	5 (2.8)
Persistence	49 (27.2)	79 (43.9)	36 (20.0)	13 (7.2)	3 (1.7)
Accuracy, attention to detail	47 (26.1)	71 (39.4)	51 (28.3)	10 (5.6)	1 (0.6)
Planning, coordinating and organizing	66 (36.7)	79 (43.9)	31 (17.2)	3 (1.7)	1 (0.6)
Loyalty and integrity	56 (31.1)	83 (46.1)	31 (17.2)	9 (5.0)	1 (0.6)
Getting personally involved in OH activities	93 (51.7)	63 (35)	22 (12.2)	2 (1.1)	0 (0.0)
Adaptability	64 (35.6)	78 (43.3)	32 (17.8)	4 (2.2)	2 (1.1)
Work ethics and integrity	63 (35.0)	77 (42.8)	26 (14.4)	14 (7.8)	0 (0.0)
Professional approaches to solving workplace challenges	56 (31.1)	74 (41.1)	37 (20.6)	11 (6.1)	2 (1.1)

### Application of one health competencies in navigating the job market

The majority of respondents, 78.6% (143/182) had current jobs that required the application of OH knowledge and skills. Almost all, 95.6% (174/182) of the respondents learnt employable skills, which enabled them in navigating the job market. The most cited employable skills were: teamwork 84.1% (153/182), communication skills, 80.8% (147/182) community engagement 76.9% (140/182). The majority, 89.6% (163/182) of respondents reported that they utilised OH competencies in the search for employment. Of these, 54.9% (100/182) applied community engagement while in search of employment, 35.2% (64/182) applied community entry, project planning and management, 73.6% (134/182) applied communication skills, and 49.5% (90/182) applied report writing (Table 3).

**Table 3:** Application of OH competencies in navigating the job market by the 2013–2018 AFROHUN-Uganda alumni

OH competencies	Category	*N* = 182 (n) %
Current job requires application of OH knowledge and skills	Yes	143 (78.6)
	No	16 (8.8)
	Have never got employed	23 (12.6)
Learnt any employable skills from the OH activities	Yes	174 (95.6)
	No	8 (4.4)
Employable skills learnt by the respondents^a^	Community engagement	140 (76.9)
	Community entry	120 (67)
	Project planning and management	87 (47.8)
	Report writing	120 (65.9)
	Data analysis	72 (39.6)
	Communication skills	147 (80.8)
	Problem-solving skills	117 (64.3)
	Teamwork	153 (84.1)
	Other skills	10 (5.5)
Utilised OH competencies in the search for employment	Yes	163 (89.6)
	No	19 (10.4)
Employable skills applied by the alumni while in search for employment^a^	Community engagement	100 (54.9)
	Community entry	64 (35.2)
	Project planning and management	64 (35.2)
	Report writing	90 (49.5)
	Data analysis	47 (25.8)
	Communication skills	134 (73.6)
	Problem-solving skills	89 (48.9)
Attended an interview for any job after participating in the AFROHUN-Uganda OH activities	Yes	146 (80.2)
	No	36 (19.8)
Skills required during their most recent job interview^a^	Community engagement	72 (39.6)
	Community entry	43 (23.6)
	Project planning and management	48 (26.4)
	Report writing	64 (35.2)
	Data analysis	54 (29.7)
	Communication skills	101 (55.5)
	Problem-solving skills	84 (46.2)
	Teamwork	104 (57.1)
Qualified for the job	Yes	129 (70.9)
	No	17 (9.3)
	Did not attend any interview	36 (19.8)

^a^Multiple responses

### One health competencies required in the alumni’s workplaces

To a very high extent about 21.7% (34/157) required OH field-specific theoretical knowledge at their workplaces, 27.4% (43/157) required OH field-specific practical knowledge/skills, 42.7% (67/157) required a change in attitude and perceptions towards working with people from different disciplines, 49.0% (77/157) required collaboration and networking skills, and more than half, 51.0% (80/157) required team-building skills (Table 4).

**Table 4:** Extent of Application of OH competencies at workplaces by the 2013–2018 AFROHUN-Uganda alumni

	Extent of application of OH competencies *N* = 157 (n)%				
OH competencies	Very high	High	Some	Limited	Not at all
OH field-specific theoretical knowledge	34 (21.7)	67 (42.7)	38 (24.2)	16 (10.2)	2 (1.3)
OH field-specific practical skills	43 (27.4)	63 (40.1)	38 (24.2)	11 (7)	2 (1.3)
Attitudinal change towards working with multi-disciplinary teams	61 (38.9)	67 (42.7)	22 (14)	7 (4.5)	0 (0.0)
Critical thinking skills	67 (42.7)	56 (35.7)	30 (19.1)	4 (2.5)	0 (0.0)
Creativity skills	63 (40.1)	62 (39.5)	28 (17.8)	3 (1.9)	1 (0.6)
Collaboration and networking skills	77 (49)	58 (36.9)	17 (10.8)	5 (3.2)	0 (0.0)
Communication skills	95 (60.5)	50 (31.5)	10 (6.4)	2 (1.3)	0 (0.0)
Leadership skills	69 (43.9)	63 (40.1)	20 (12.7)	5 (3.2)	0 (0.0)
Social (influencing) skills	63 (40.1)	60 (38.2)	29 (18.5)	5 (3.2)	0 (0.0)
ICT skills	67 (42.7)	56 (35.7)	22 (14)	8 (5.1)	4 (2.5)
Analytical skills	62 (39.5)	65 (41.4)	24 (15.3)	6 (3.8)	0 (0.0)
Problem-solving skills	77 (49)	61 (38.9)	18 (11.5)	1 (0.6)	0 (0.0)
You are able to take initiative	58 (36.9)	67 (42.7)	27 (17.2)	5 (3.2)	0 (0.0)
Entrepreneurial skills	32 (20.4)	39 (24.8)	52 (33.1)	25 (15.9)	9 (5.7)
Team building skills	80 (51)	54 (34.4)	20 (12.7)	3 (1.9)	0 (0.0)
People management skills	78 (49.7)	55 (35)	24 (15.3)	62 (39.5)	0 (0.0)
Customer orientation skills	53 (33.8)	26 (16.6)	12 (7.6)	4 (2.5)	0 (0.0)
Assertiveness	55 (35)	57 (36.3)	40 (25.5)	3 (1.9)	2 (1.3)
Decisiveness	65 (41.4)	60 (38.2)	25 (15.9)	5 (3.2)	2 (1.3)
Persistence	71 (45.2)	58 (36.9)	23 (14.6)	4 (2.5)	1 (0.6)
Accuracy and attention to detail skills	79 (50.3)	62 (39.5)	11 (7)	5 (3.2)	0 (0.0)
Planning, coordinating and organizing skills	84 (53.5)	54 (34.4)	15 (9.6)	4 (2.5)	0 (0.0)
Loyalty and integrity skills	90 (57.3)	51 (32.5)	14 (8.9)	2 (1.3)	0 (0.0)
You are required to get personally involved into activities	81 (51.6)	64 (40.8)	9 (5.7)	3 (1.9)	0 (0.0)
Adaptability skills	66 (42)	63 (40.1)	22 (14)	5 (3.2)	1 (0.6)
Work ethics and integrity	95 (60.5)	50 (31.8)	11 (7)	1 (0.6)	0 (0.0)
You provide professional solutions to your workplace challenges	69 (43.9)	64 (40.8)	21 (13.4)	3 (1.9)	0 (0.0)

## Discussion

This study explored the extent of acquisition and application of the one health competencies among AFROHUN-Uganda OH alumni. The study revealed that majority of the alumni had jobs that required the application of OH knowledge, skills, and competencies. Alumni were assessed for skills in community entry and engagement, project planning and management, data analysis and report writing, communication, problem-solving, and teamwork during their most recent job interviews. More than three-quarters of the alumni acquired field-specific theoretical knowledge and practical skills. In addition, a significant proportion reported a change in attitude and perceptions towards working with individuals from other disciplines.

The current study revealed that almost all the alumni learnt employable skills from OH activities. The skills reported by the alumni included community entry and engagement, applied communication skills, project planning and management, and report writing. The alumni pointed out that they applied the different skills while in search of employment opportunities. Just like Kolb’s experiential learning cycle [41, 42], AFROHUN-Uganda OH activities such as the One Health Institute (OHI) and the graduate placement incorporate experiential learning that is intended to enable students to “learn by doing” and by reflecting on their experience, while under supervision. The OH experiential learning implemented by AFROHUN-Uganda can stimulate academic inquiry by promoting interdisciplinary learning, collaboration, civic engagement, career development, cultural and gender sensitivity, and leadership [41–45]. Experiential learning also improves the quality of personal involvement, i.e. the whole person in both his feeling and cognitive aspects being in the learning event [46–48]. The OH experiential learning process implemented by AFROHUN-Uganda is largely student-led and involves participatory and interactive learning, reflection, abstract conceptualization, and active experimentation [49, 50]. This mode of training helps the students, herein referred to as the alumni, to translate the practical knowledge and skills acquired during their training. The knowledge, skills and competencies acquired from the OH field experiential learning activities can be applied by the alumni at their workplaces, and in solving global health challenges. Other than our study, several have also reported how experiential learning stimulates academic inquiry and enables global health practitioners to acquire practical skills and experiences through practical tasks [51, 52]. Some of the other institutions which apply experiential learning to build the capacity of public or global health practitioners are documented by Rodríguez, Jessani [53].

The skills gained by the alumni in the current study have also been emphasized or reported among other OH or global health training programs. Knowledge, skills and competencies reported in our study also form the basis of the one health or global health training conducted by the Southeast Asia One Health University Network [30], Conservation and Ecosystem Health Alliance [54], One Health Network South Asia [55], the EcoHealth Alliance [56] and the AFYA BORA consortium fellowship, a global health training program implemented in Botswana, Cameroon, Kenya, Tanzania, and Uganda [57, 58]. Available evidence indicates that alumni who engaged in these training programmes acquired skills in leadership, communication, team building, strategic planning, project management, ethics, and monitoring and evaluation [59]. Just like the alumni in the AFROHUN-Uganda training programme applied OH knowledge, skills and competencies, those in the AFYA BORA consortium fellowship reported that they would apply the knowledge, skills and competencies gained through their experiential placement at their workplaces [59]. Both the AFROHUN-Uganda training and AFYA BORA consortium fellowship demonstrated that experiential learning is critical for imparting knowledge, skills and competences, despite having different target groups. The AFYA BORA consortium fellowship targets applicants with a postgraduate qualification in medicine or nursing, or public health professionals with a Doctoral degree in public health or a related field [57]. On the contrary, AFROHUN-Uganda targets heterogeneous applicants with at least a bachelor or masters’ degree [1].

Our study revealed communication, teamwork, collaboration, community engagement, and problem-solving as some of the skills required from the alumni during job interviews. These skills are part and parcel of the OH training offered by AFROHUN to students participating in OH activities, including the OHI, outbreak investigation and graduate placement [1]. The communication skills learnt by the alumni included listening, nonverbal communication, clarity and concision, and friendliness among others. Such skills are essential in today’s workplaces since they improve customer care and the image of the employer/organisation [60, 61]. The AFROHUN communication module also focuses on risk communication, application of behaviour change, communication theories, critical thinking, communication strategies, social styles and social awareness. Risk communication is often at the centre of tackling global health challenges such as transboundary infectious disease outbreaks such as EVD and COVID-19 [62–65]. Effective communication skills allow global health practitioners to explore different communication strategies or approaches, and the risks of transboundary communication and foster collaborations in the event of a global health challenge [66, 67]. The role of effective communication as a key competence in global health is also emphasized in a systematic review by Sawleshwarkar and Negin [68], and other global health training programs [30, 69, 70].

The current study also revealed teamwork as a skill required by employers during job interviews. Teamwork is known to improve the employee’s performance. Employees in organisations where teamwork is embraced are more likely to relate better since they have an opportunity to bond with one another. Similarly, tackling global health challenges is reliant on team effort. There is evidence that global health solutions such as the development and supply of vaccines depend on a team effort of scientists, governments, science administrators, technicians, and those involved in the supply chain [71]. Besides, teamwork fosters an effective response to global health crises [72]. Several global health training programs incorporate teamwork as a key competence to solving transboundary challenges [30, 58, 73]. Given the critical role played by teamwork, it was exercised even amidst the COVID-19 restrictions in transboundary movement by the use of innovative solutions such as virtual teams [74, 75]. Without teamwork, therefore, the attainment of global health goals and targets remains futile.

It is worth noting that community engagement and entry was a critical skill learnt and applied by the alumni. Community engagement and entry is a critical skill in many workplaces as it involves building a strong partnership between community-based organisations or institutions and individuals with the collective vision of benefiting the community/target population. This explains why most organisations required alumni with community engagement skills during the interviews and the reason why nearly three-quarters of the alumni qualified for the respective jobs. In the context of a global health crisis, engaging the community builds trust, and increases ownership and sustainability of interventions. Mugisha, Travis [54] report community entry and engagement as critical OH competencies. A rapid synthesis by Gilmore, Ndejjo [76] revealed that skills in community engagement can contribute to the development of contextually appropriate interventions, and facilitate community entry, trust, behaviour change communication, surveillance and contact tracing, and logistical support during a global health crisis.

More than three-quarters of the alumni enrolled on the study agreed that to a high extent they acquired field-specific theoretical knowledge and practical knowledge/skills from the OH training programmes. The mode of training employed by AFROHUN involves in-class and virtual sessions which are vital in imparting theoretical knowledge to the alumni. The training also involves field experiential learning which involves students being placed in organisations or institutions that have a vast interest in using the OH approach in tackling global health challenges. Experiential learning involves students working with the local communities in identifying community challenges and innovatively developing solutions to the identified health challenges [49]. Through experiential learning, students are also given an opportunity to develop innovations and apply for small grants. These avenues help to impart theoretical and practical knowledge and skills. Experiential learning has been shown to improve practical knowledge and skills [77–79], while in-class and virtual sessions are known to improve theoretical knowledge among students [80–82]. Our findings are not different from those of Mugisha, Travis [54], which indicated experiential learning as central to the success of their one health training program in Kasese district, western Uganda.

A significant proportion of the alumni in the current study agreed that participation in the AFROHUN-Uganda OH activities was instrumental in facilitating their change in attitude and perceptions toward working in multidisciplinary teams. The goal of the OH training programs is to foster multi-sectoral collaboration and transdisciplinary. To achieve this goal, AFROHUN-Uganda equips students with knowledge on management, leadership, culture and ethics, communication, systems thinking, and collaboration that are vital in influencing students’ attitudes and perceptions towards working with individuals from other disciplines. This finding illustrates the positive stride made by AFROHUN-Uganda in influencing positive behaviours towards collaboration among the different cadre and sectors. A change in attitude and perceptions is widely documented as one of the facilitators of the application of the OH approach [83, 84]. There is evidence that OH interventions aim to improve the attitude of a new generation of scientists and problem solvers in order to work together as they tackle global health challenges [85].

More than three-quarters of the alumni in the current study agreed that, to a high extent, they had acquired leadership skills. Nearly three-quarters of the alumni mentioned that their jobs required leadership skills. Attainment of optimal health at the environment-human-animal nexus requires effective leadership. In order to achieve the objectives of the OH approach, it is important that those trained and their colleagues have adequate leadership and management skills such as active listening, empathy, and strategic thinking. Effective leadership skills enable managers to inspire a vision and motivate fellow employees towards achieving OH goals. It is also critical in enabling managers in empowering team members to work at their full potential and to take responsibility for decision making. Besides, effective leadership is critical for building consensus among disparate sectors and fostering champions for cohesion and change [86]. Other roles of leadership in advancing OH are documented by Amuasi, Lucas [86] and Zinsstag, Schelling [87]. Due to the critical role played by leadership capacity in addressing global health challenges, it has been incorporated in several trainings or fellowships aimed at improving prevention and response to global health problems [30, 54, 58].

### Study limitations

This being a quantitative study, we could not document in sufficient detail how the OH competencies acquired were applied to solve global health challenges. Thus, we recommend a qualitative study to further this research question. We also used a self-administered e-questionnaire which is subject to social desirability bias [88, 89]. Despite these limitations, our study provides valuable insights into the extent of acquisition and application of health approaches in solving global health challenges.

## Conclusion

Our study revealed that the majority of the OH alumni had acquired jobs that required the application of OH knowledge and skills such as teamwork, effective communication, community entry and engagement, report writing and problem-solving skills. The competencies acquired by the OH alumni were to a high extent applied at their workplaces. Notably, the OH activities to a high extent changed the attitude of the alumni towards working in multi-disciplinary teams. This study revealed the significant contribution of the AFROHUN Uganda OH activities towards supportive work environments and highlights areas of improvement such as supporting the trainees to acquire people-management skills, innovation, and an entrepreneurial mindset.

## Data Availability

The datasets analysed during the current study are available from the corresponding author upon reasonable request.

## Abbreviations

AFROHUN: Africa One Health University Network
EVD: Ebola Virus Disease
MUST: Mbarara University of Science and Technology
OH: One Health
OHCEA: One Health Central and Eastern Africa
OHI: One Health Institute
STF: Systems Theory Framework
USAID: United States Agency for International Development
